# Person-environment interactions shape maternal perceptions of stress and coping during pregnancy and postpartum: a qualitative analysis

**DOI:** 10.1186/s12889-026-28601-9

**Published:** 2026-07-29

**Authors:** Kayla D. Longoria, Tien C. Nguyen, Sara Dube, Frances Cates, Oscar Y. Franco-Rocha, Nicole Manley, Sreya Gandra, Michelle L. Cassidy, Elizabeth M. Widen

**Affiliations:** 1 School of Nursing, University of Texas at Austin, Austin, TX USA; 2 Department of Physiological Nursing, School of Nursing, University of California, San Francisco, USA; 3 College of Natural Sciences, University of Texas at Austin, Austin, TX USA; 4 School of Medicine, University of Pittsburgh, Pittsburgh, PA USA; 5 Department of Nutritional Sciences, The University of Texas at Austin, Austin, TX USA; 6 College of Liberal Arts, University of Texas at Austin, Austin, TX USA; 7 Department of Women’s Health, Dell Medical School at The University of Texas at Austin, Austin, TX USA; 8 Department of Pediatrics, The University of Texas at Austin Dell Medical School, Austin, TX USA

**Keywords:** Maternal stress, Social determinants of health, Maternal well-being

## Abstract

**Background:**

Maternal stress during pregnancy and postpartum is consistently associated with increased risk for maternal, obstetric, and offspring health complications, highlighting its transdiagnostic relevance for maternal and child health. While stress associated with major life events and trauma (e.g., intimate partner violence) is well-studied, comparatively far less attention has been paid to understanding how routine perinatal demands are experienced within the contextual realities of pregnancy and postpartum and how these experiences shape maternal perceptions of stress and coping across these two distinct yet interrelated periods.

**Methods:**

Data were obtained from a subset of participants (*n* = 17) enrolled in a larger prospective cohort study. Semi-structured interviews during pregnancy (*n* = 10) and postpartum (*n* = 15) were conducted remotely (i.e., Zoom, phone). Interviews were independently analyzed using inductive thematic analysis, respective to each perinatal period.

**Results:**

Resource accessibility emerged as an overarching theme, with five core themes acting as barriers or facilitators to mental well-being. Three resources–social support, logistics, and physical health–were considered influential across both perinatal periods, to varying degrees. When resources were inaccessible during pregnancy, they became barriers to mental well-being, contributing to experiences of loneliness, frustration, worry, an increased mental load, and ultimately, increases in perceived stress. These experiences often intensified postpartum due to unmet expectations of having a higher level of support to manage increases in logistical demands (e.g., domestic labor, caregiving) while simultaneously navigating postpartum recovery and workforce participation. Conversely, with higher resource access, mother’s perceived ability to cope with stress arising from routine perinatal demands, the COVID-19 pandemic, and other logistical responsibilities increased.

**Conclusions:**

Findings indicate routine perinatal demands are perceived to shape maternal experiences of stress and coping during pregnancy and postpartum, emphasizing the importance of resource accessibility and contextual determinants in shaping maternal well-being. Future research is needed to build on these findings and highlight the need for multilevel interventions to increase maternal support and resources to manage cumulative perinatal demands, which can ultimately improve health trajectories for women, children, and families.

## Background

Maternal mental health is a critical public health issue, as complications during the perinatal periods (pregnancy, postpartum) can adversely affect physical and mental health trajectories of women, children, and families [1]. The precise causes of these complications are not well defined; however, evidence across diverse geographic and socioeconomic contexts consistently indicates stress as a significant contributor [2–4]. While stress associated with major life events (e.g., bereavement) and trauma (e.g., intimate partner violence) is well-studied, comparatively far less attention has been paid to understanding how women experience routine perinatal demands (e.g., physical symptoms, caregiving, identity shifts, navigating prenatal appointments with occupational responsibilities) as stressful or manageable within the contextual realities of their lived experiences during pregnancy and postpartum [5–7]. Specifically, critical gaps remain in understanding how contextual factors simultaneously operate across multiple levels, influencing mother’s experiences of routine perinatal demands and perceptions of stress and coping, both within and between these two distinct perinatal periods.

Maternal stress is recognized as a multidimensional, context-dependent phenomenon that involves physiological and psychological responses to real or perceived threats that can overwhelm an individual’s capacity to cope [8]. Individuals differ considerably in how they appraise and respond to similar demands because stressors rarely occur in isolation, and perceptions of manageability depend on the broader constellation of multiple competing demands and the social and structural conditions in which they are experienced [8]. The recent COVID-19 pandemic offers a naturally occurring example of these dynamic interactions, highlighting globally how changes in social and structural conditions can fundamentally alter individual experiences, appraisals, and the capacity to cope with everyday demands. Thus, maternal stress and coping may be better understood as co-constructed experiences emerging from dynamic interactions between individuals and their broader social and structural conditions (person-environment interactions) [5, 9].

Contextual determinants are neither static nor experienced in isolation; rather, they often co-occur, evolve over time, and interact across multiple levels (e.g., individual, organizational, community, policy) [10]. Such complexities present significant methodological challenges in quantifying their impact on individual experiences [11, 12]. Consequently, comprehensive examinations of these contextual factors aimed at understanding how they influence women’s experiences of routine perinatal demands and perceived ability to cope remains a critical yet underexplored area of public health research [13, 14]. Additionally, maternal stress research has focused heavily on clinical (e.g., depression diagnosis or clinically significant symptoms) and high-risk groups (e.g., clinical and sociodemographic characteristics) [14, 15]. While this work remains essential, it limits understanding of how stress is experienced across the broader perinatal population, reducing opportunities for early detection and intervention before cumulative demands begin to exceed available resources to cope and progress to health and mental health complications. Examining maternal stress in categorically low-risk groups may help identify underrecognized yet modifiable stressors that are relevant to the broader population and highlight contextual determinants that may enhance intervention uptake outside of controlled research environments. Such insights are critical for identifying needs of the broader perinatal population and informing population- or policy-level strategies to increase support for pregnant and postpartum individuals amid this dynamic life-stage [16, 17].

Qualitative approaches offer a critical opportunity to understand contextual and subjective dimensions of stress through the lived experiences of pregnant and postpartum women [18], deepening understanding of how perinatal women experience, interpret, and manage stress during each perinatal period and across the perinatal continuum. The purpose of this qualitative study is to explore the perspectives of pregnant and postpartum women to understand how they experience and navigate the demands of pregnancy and postpartum, and how these demands are experienced as stressful or manageable within their broader social and structural conditions. By focusing on a low-risk sample, this study can provide contextually grounded insight into upstream determinants of maternal stress and coping, generating evidence to inform future research and identify opportunities for population-level strategies to promote maternal well-being outside of clinical-specific paradigms.

## Methods

This is a secondary analysis using data collected between 2020 and 2021 from a subset of participants (*n* = 17) enrolled in an ongoing prospective cohort study (NCT04132310). Design and recruitment procedures for the parent study have been reported in detail elsewhere [19]. Briefly, participants were recruited in early pregnancy (< 16 weeks gestation), primarily from clinics within two commonly utilized hospital systems in Central Texas. Roughly 6 months after parent studyenrollment began, the World Health Organization declared COVID-19 a pandemic (March 2020) [20], resulting in significant disruptions in research operations of the parent study. In response to the rapidly evolving global pandemic, qualitative data collection was added to the parent study to capture participant’s experiences during pregnancy and postpartum, including but not limited to, how they experienced and navigated changes in employment, healthcare, social support, and food access. This study was approved and monitored by the Institutional Review Board at The University of Texas at Austin (2018-05-0127).

Data were collected using a semi-structured interview guide consisting of open-ended questions (Table 1). Demographic data were collected via self-report questionnaires in REDCap. Between June 2020 – October 2020, two study personnel trained in qualitative methods (SD, GD) completed a total of 25 in depth interviews with 17 perinatal women either by phone (*n* = 23) or Zoom (*n* = 2). Each interview was audiotaped and had two interviewers (SD, GD). Of the 25 interviews, 10 were from pregnancy and 15 were from postpartum. Eight perinatal persons completed two interviews, one from each perinatal period. Interviews were conducted in English and lasted roughly one to one and a half hours. Details on audio file transcription, review, and de-identification have been previously reported [19].

**Table 1:** Semi-structured interview guiding qualitative data collection in parent study

	**Semi-structured interview questions**
1	Can you describe how the COVID-19 pandemic affected your health during and after your pregnancy?
2	Can you describe how your eating behaviors changed during the pandemic?
3	Can you describe how the pandemic changed your activity levels?
4	Can you describe how the pandemic changed your sleeping habits?
5	What types of emotion did you feel as you became more aware of the COVID-19 pandemic?
6	Can you explain how the pandemic affected your healthcare?
7	What sources did you use to know what precautions to take for COVID-19 during and after pregnancy?

**Fig. 1:**
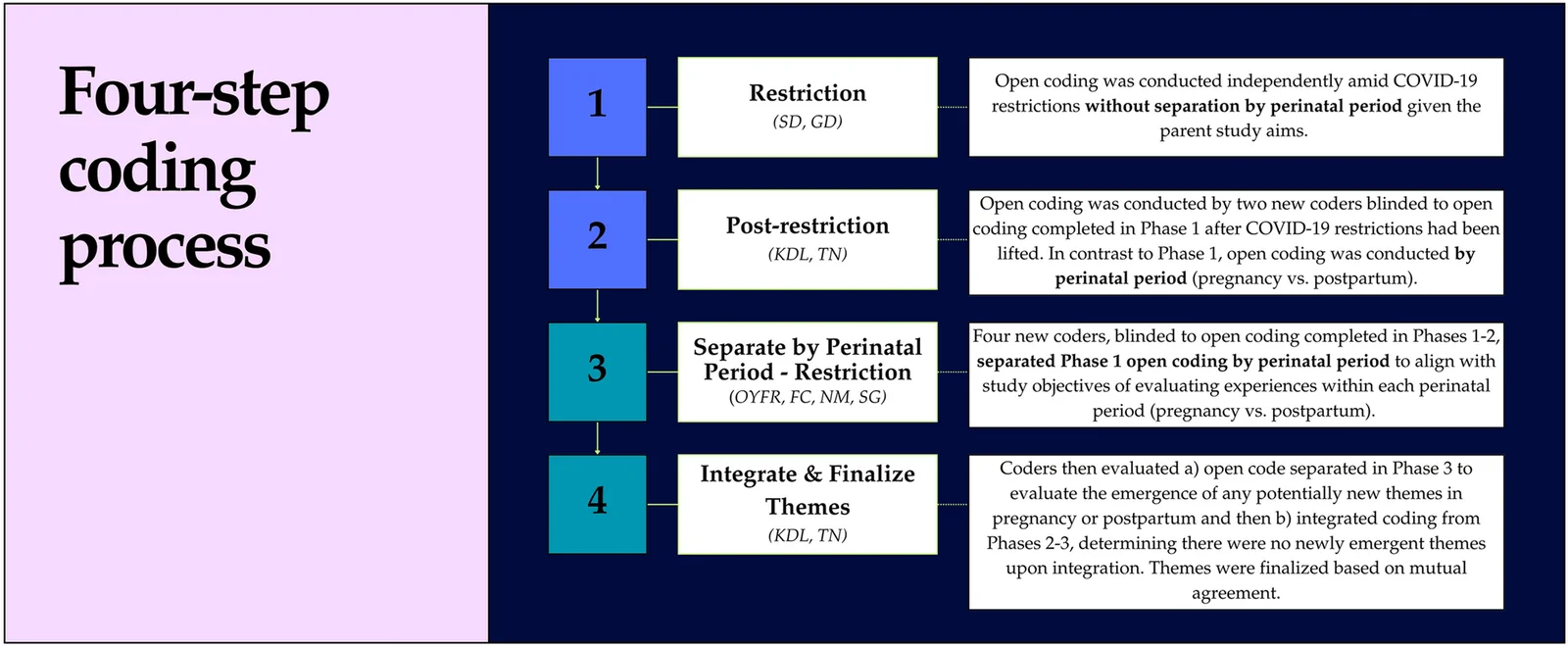
Four-phase coding process

The data were analyzed using inductive thematic analysis, guided by Braun and Clarke’s (2006) commonly used six steps: (1) familiarizing yourself with the data, (2) generating initial codes, (3) searching for themes, (4) reviewing themes, (5) defining and naming themes, and (6) producing the report [21]. A four-phase coding process was used to enhance the trustworthiness of the findings (Fig. 1).

## Results

### Participant characteristics

The mean (SD) gestational age for the pregnancy interviews was 33.89 (2.47) weeks, whereas the mean (SD) months postpartum was 2.25 (1.16). Participants were predominantly married (92%), Non-Hispanic (96%), white (88%) women delivering their first child (76%). The Beck Depression Inventory (BDI) identified 3 (30%) women experiencing elevated depression symptoms during pregnancy, with severity ranging from borderline clinical depression to severe [22, 23]. With a cut-off score of ≥ 10 [24, 25], the Edinburgh Postnatal Depression Scale (EPDS) identified 4 (27%) participants as having probable postpartum depression [26]. Additional details for participant characteristics can be seen in Table 2.

**Table 2:** Participant characteristics

Characteristics	*n*	M (SD)	%
Perinatal age			
Gestational age (weeks)	17	33.9 (2.5)	--
Postpartum (months)	17	2.3 (1.7)	--
Race			
White	14	--	82.3%
Asian	1	--	5.9%
Mixed race	2	--	11.8%
Ethnicity			
Hispanic	1	--	5.9%
Non-Hispanic	16	--	94.1%
Employment			
Full-time	17	--	100%
Education			
College graduate	9	--	52.9%
Post-graduate degree	8	--	47.1%
Partner Status			
Married	15	--	88.2%
Not married, cohabitating	1	--	5.9%
Not married	1	--	5.9%
Household income			
≤$74,999	3	--	17.6%
$75,000-$149,999	7		41.2%
≥$150,000	7		41.2%
Insurance			
Private	15	--	88.2%
Parity			
Nulliparous	11	--	64.7%
Mode of delivery			
Vaginal	12	--	70.6%
Cesarean (elective)	4	--	23.5%
Cesarean (emergency)	1	--	5.9%
Infant feeding			
Breast	15	--	88.2%
Psychiatric history			
Mood disorder	5	--	29.4%
Depression scores			
Pregnancy (BDI)^a^	17	12.7 (9.9)	--
Postpartum (EPDS)^b^	17	6.6 (5.6)	--
Perceived stress^c^			
Pregnancy	17	11.7 (6.0)	--
Postpartum	17	13.1 (8.6)	--

^a^Beck Depression Inventory (BDI-II)

^b^Edinburgh Postnatal Depression Scale (EPDS)

^c^Perceived Stress Scale (PSS)

### Themes

**Fig. 2:**
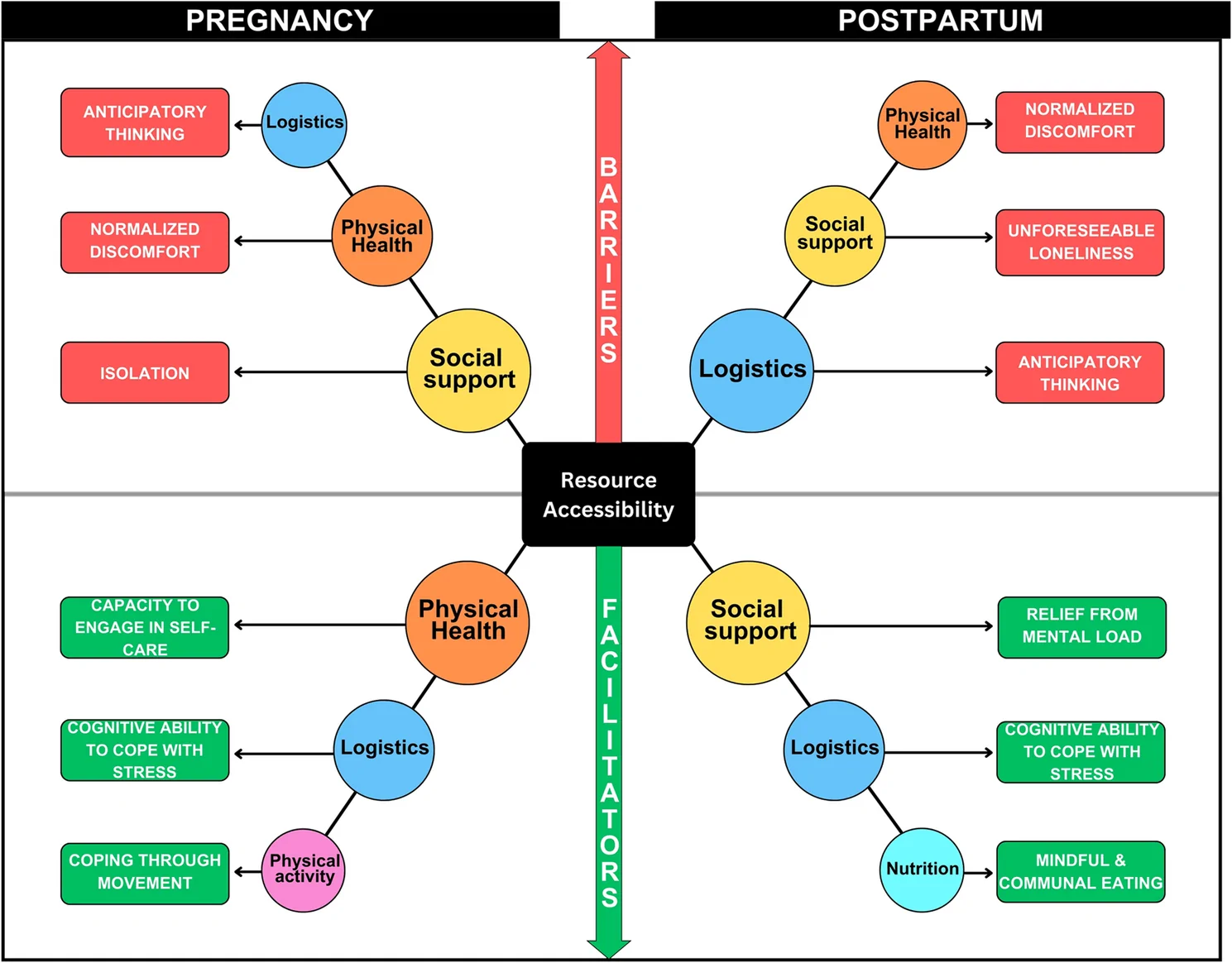
Thematic map of resources influencing mental well-being during pregnancy and postpartum

Resource accessibility emerged as an overarching theme, shaping experiences of stress and mental well-being during pregnancy and postpartum. Five themes were described as central in navigating the demands of pregnancy and postpartum: (1) social support; (2) logistics; (3) physical health; (4) nutrition; and (5) physical activity. To retain the contextual and temporal nuance of participants’ experiences, we focus on the three most salient themes across both perinatal periods (i.e., social support, logistics, physical health). Supporting participant quotes are presented in Table 3, with corresponding notations “(T#)” used throughout the text. Figure 2 presents a thematic map, organized by perinatal period and ordered by frequency, to illustrate how each resource functioned as a barrier or facilitator to mental well-being, depending on its accessibility.

**Table 3:** Direct participant quotes in support of theme descriptions

**Theme**	**T(#)**	**Description**	**Supporting quote**
Social support	T1	Need for increased support	“My 6 week one, when I was supposed to go back to be seen, I was turned away at the door because I didn’t have anyone to watch the [BABY]. I mean some of that was because, some of that was my fault because I didn’t have anybody to watch him, but I'm surely not the only single mom ya know.” *P3, postpartum*
	T2	Planning for increased support	“I had this elaborate plan of all the support I could think of and all the social contacts to avoid loneliness and help my mood.” *P12, pregnancy*
	T3	Expectations for increased support postpartum	“I had four different people who were supposed to come and stay with me at various times to help out and help me with food, and cleaning and all that stuff, and um nobody showed. So, I was alone pretty much from… a week before I gave birth.” *P2, postpartum*
	T4	Impact of lack of support on mental health	“I do think that, um, at one point I was pretty sure that I was gonna have to stop breastfeeding because I was really gonna need advanced medical care for my mental health, and I think that that was so severe because of the lack of support.” *P2, postpartum*
	T5	Social support as a social system, quality evaluated by how mutualistic relationships were perceived to be	“I think like mentally, it’s probably taking a toll because we don’t see people who want to see us and who we want to see and who are invested in our pregnancy and that we’re close with.”* P1, pregnancy*
	T6	Perceptions of virtual interactions	“Not the worst, but it is not the same either.”*P12, pregnancy*
	T7	Continuity of care in building patient-provider relationship	“I had a new doctor every single time. I've just been kind of floating around in it. And I think not being able to see these people in person hasn’t given me – I’m just going through this unknown transformative process."* P9, pregnancy*
	T8	Inherent trust in labor and delivery trained healthcare providers	“Do whatever the doctors and nurses say.” P15,* pregnancy*
	T9	Ask partner for help or provide instruction	“Ask for it [help] and teach him [partner].” P8*, postpartum*
	T10	Desire for partner to be proactive in parenting and anticipating needs of the dyad	“Want [PARTNER] to be actively parenting.”* P4, postpartum*
	T11	Mental load induced stress	“And sometimes what he was able to do was more stressful than helpful." *P3, postpartum*
Logistics	T12	Healthcare appointments	“We did have all of my prenatal visits set up until delivery date, and all of those were cancelled. Actually, without my knowledge until I got to the front desk to say okay my next appointment is this time on this day and they said well actually we cancelled all of your appointments.” *P11, pregnancy*
	T13	Adjusting dietary habits	“Actually, just within the last week, I have started to cut dairy. Which I was never a big, I don’t know if I want to say believer in like food allergies in infants and reactions, but we had a horrible week last week of Colic or reflux. I've never had to cut food, I've always just ate what I want to eat.” *P16, postpartum*
	T14	Insurance	“The insurance companies were cutting off the telehealth. We have to pay like an additional fee now to continue doing those. So, I don’t know if that’s going to impact me just because I'm getting so close that they might need me to come in.” *P10, pregnancy*
	T15	Workforce participation	“Going back to work and all that are the biggest emotional rollercoasters.” *P1, postpartum*
	T16	Strategizing paid time off (PTO)	“When they [PROVIDER] were only offering morning appointments, that was like a big barrier just having to like – ya know I'm trying to save up my time so I can take as much of that leave when the baby’s born.” *P15, pregnancy*
	T17	Health related information	“Some of the like apps that I was using to track my pregnancy, like “What to Expect”. Um, they send out little emails like every few days about different topics.” *P6, postpartum*
	T18	Planning for childbirth and care	“Then becoming more educated on it made me like “okay well, maybe I don’t feel comfortable with ya know – I don’t want to be induced at 39 weeks” and that was just like the go to. I don’t like that or – not that anyone is gonna make me do it. But, ya know, if I had an uncomplicated birth process and don’t need to be in one (cesarean section), then maybe that’s a better decision to make for the health of me and my baby.” *P12, pregnancy*“Figuring out like how we could manage like caring for both kids was like incredibly stressful and like time consuming.” *P8, postpartum*
	T19	Planning for increased support postpartum	“I had this elaborate plan of all the support I could think of and all the social contacts to avoid loneliness and help my mood.” *P3, postpartum*
	T20	Increased need to plan ahead	“I've kind of made preparations for realizing I need to pack an extra change of clothes, because I have to pick her up after work, and I'm exposed to 200 plus kids a day. So, um just like those kind of things. Taking longer and thinking about that further ahead.” *P11, postpartum*
	T21	Support from social network increasing efficiency	“Yeah, pretty much. I wasn’t limited to anybody’s hours. There was no traffic to fight to get there, so it was something that could be done, and get up and go, and pretty much do it whenever I have the time. And some days it was better to go in the mornings, some days it was easier to do it in the late evenings. Um, but because of the that, things were definitely more efficient.” *P7, postpartum*
	T22	Increased time for self-care when support is available	“I have really enjoyed being able to get out and walk. Um, like I'm trying to walk like twice a day. So, I'm walking right when we’re done with work, we take a walk as a family. And then most nights I'm walking by myself after we put [OLDER CHILD] to bed, which is just kind of like my quiet me time.” *P10, postpartum*
	T23	Schedule flexibility	“I have appreciated the ability to be able to work from home, just with the complications I had developed towards the end of my second trimester and now, just because navigating a [work environment], I don’t know how I would’ve done that.”* P12, pregnancy*
	T24	Balancing childcare and a full-time job	“Given the fact that part of my working day is taken up by childcare, it's probably more acute with the pandemic and I'm probably staying up later than I would otherwise.” *P15, pregnancy*
	T25	Sacrificing personal needs	“I feel a sense of hopelessness that it’s never going to get better and that my worries of not getting to have a life are totally true now because I have no life now other than being at home and caring for this baby.” *P1, postpartum*
	T26	Inherent duties of being a mother	So, it’s kind of a lot more responsibility of caring rests on me, as the mother is going to naturally be the one going into the appointments because I had to answer questions, I had to take the postpartum things. I’m sure [PARTNER] could be the one but doesn’t make sense for him. It’s been more emotionally burdensome.”* P2, postpartum*
	T27	Beliefs reinforced	“All those [in-person OB appointments] have been normal except for the fact that my husband can’t come, so I’ve gone to all of them by myself.” *P17, pregnancy*“I have been to the doctor more for [BABY] than I have myself.” P9*, postpartum*“My husband doesn’t know where to go, and I really wish that he could be more prepared... [where to go once pregnant person goes into labor].” *P14, pregnancy*
	T28	Increased support can remove logistical barriers and provide relief from the mental load	"But I wish [EMPLOYER] would give me time to pump [laughs] because then I would be less stressed about it. You never want to like, ya know, you just don’t wanna be that person. But I just am gonna have to be that person. I certainly don’t want to be on the bad list with my bosses."* P2, postpartum*
Physical health	T29	Health concerns focused on others	“My biggest worry there is you know picking something up and then bringing it back home to someone in my family.” *P16, pregnancy*
	T30	Physiological demands of pregnancy	"I think I’ve just had a really tough time being pregnant physically. I’ve just like had a lot of swelling. I’ve been in a lot of pain and discomfort." *P8, pregnancy*
	T31	Ability to engage in self-care	“I took up gardening, which I've never done in my life. I think it's kind of helping. It forces me to go outside under the sun I suppose.” *P17, pregnancy*
	T32	Fear of contracting COVID-19 and implications for others and self	“I think I'm feeling a little more stress as we get closer to like, I'm really terrified of going to the hospital. Like I'm educated enough that I feel like that’s the best decision, but I'm scared to go to the hospital… and come out with COVID [laughs]."* P10, pregnancy*
	T33	Positive postpartum experience due to physical health being prioritized	“And just was very happy to see that there are doctors out there that are starting to acknowledge that you can go to physical therapy – ya know there can be lasting effects on your body from having a baby obviously. But, that there are physical therapists who are trained to help you get your pelvic floor back in shape and fix diastasis recti if your abdomen has split. Ya know, like all those little things that it just seemed like people kind of put up with after labor and delivery, but they don’t have to. So anyways, I had a really wonderful postpartum experience.” *P15, postpartum*
	T34	Normalized psychological challenges	“Those first two weeks after birth I was like really up and down in terms of emotions. Like obviously super excited to have [BABY] at home and to be [unintelligible] with her and stuff but also like I felt really, I just cried a lot and was getting a lot – I would think about ways that she could get hurt and stuff and that would create anxiety. So, it was very much like a rollercoaster in the first couple of weeks, which the nurse I talked to said was normal. So like okay, that doesn’t seem normal, but okay.” *P4, postpartum*
	T35	Challenges of physical symptoms of pregnancy	“Like, my joints and muscles are like separating, so I have to wear- if I'm walking for a while I'm supposed to wear a back brace thing and I'm swelling pretty bad so I'm supposed to be off my feet a lot more.”* P14, pregnancy*
	T36	Being on high alert	“The ability to turn off my brain as much” *P2, postpartum*
	T37	Sleep quality based on child’s temperament/development	“[BABY] is pretty efficient, so thankfully – like my [OLDER CHILD] used to nurse for 40 minutes or an hour. And [BABY] is like super quick. So, I feel like we are up less.” *P1, postpartum*
	T38	Not enough time for basic needs	"It's like, do I sleep or do I eat? Or do I eat or do I get dressed? These are my choices.”* P9, postpartum*
	T39	Not enough time for self-care or health promotion strategies	“There’s just not time for me.”*P11, postpartum*

#### Theme 1: Social support

Low social support was the primary barrier to mental well-being during pregnancy and the second leading barrier postpartum. While social support ranked fourth in promoting mental well-being during pregnancy, it was seen as a foundational resource for enhancing access to the three primary facilitators (i.e., physical health, logistics, physical activity). In contrast, during the postpartum period, social support was the most important resource in promoting mental well-being. Perinatal individuals described feelings of loneliness when social support was unavailable or inaccessible. Even when social support was limited during pregnancy, many mothers anticipated it would increase postpartum to match the heightened demands of recovery, newborn care, and balancing occupational and domestic labor responsibilities (T1). When support did not materialize, the loneliness they experienced was not only unexpected but also elevated (T2, T3, T4). Conversely, when social support was perceived as adequate, they felt relief from the mental load associated with perinatal demands.

Social support was described as a network of dynamic relationships (e.g., partner, family, friends, healthcare providers, employer/colleagues). The quality of these relationships were evaluated on the perceived level of mutualism (T5). Relationships high in mutualism provided a sense of interpersonal and intrapersonal stability, reducing worries and loneliness as mothers navigated the “unknowns” of the perinatal period and the COVID-19 pandemic. In-person interactions were preferred, but virtual interactions were considered acceptable (T6). For healthcare, comfort with virtual care depended on individual needs and the strength of the patient-provider relationship. Continuity of care was viewed as important for building trust and strengthening the relationship (T7). Although pregnant individuals faced challenges with consistent support from prenatal providers, they expressed trust in their labor and delivery-trained healthcare providers, which was reflected in their birth plans (T8).

COVID-19 restrictions limited in-person interactions largely to partners, increasing reliance on partner support throughout the perinatal period. During pregnancy, partner support was often seen as inadequate when partners failed to take a proactive role in managing logistical responsibilities and preparing for the baby’s arrival. Postpartum, women expected greater partner involvement due to added stressors associated with recovery, infant care, and balancing work and domestic responsibilities. However, many reported partner support remained low after birth. Having to direct partners in caregiving or household tasks (T9) led to feelings of frustration, anger, and resentment, as postpartum women expected shared responsibilities without prompting, anticipating their partner would adapt to the postpartum demands as they had to (T10). The burden of delegation compounded the mental load and emphasized perceived imbalances in shared responsibilities (T11).

#### Theme 2: Logistics

High logistical demands was the second leading barrier to mental well-being during pregnancy and the primary barrier postpartum. Low logistical demands, however, was the second leading facilitator to mental well-being during both perinatal periods. Pregnancy-related logistical stressors included managing frequent healthcare appointments (T12), adjusting and monitoring dietary intake (T13), navigating health insurance (T14), limited paid time off and other work-related stressors (T15, T16), seeking health information for the mother-child dyad (T17), and preparation for childbirth and newborn care (T18). Postpartum, logistical stressors intensified to also include newborn care and feeding, physical recovery, limited paid maternity leave, childcare, and an overall increase in planning for and completing activities both within and outside of the home (T19). Logistical demands associated with the perinatal periods contributed to a cycle of anticipatory thinking, and when these demands became overwhelming or seemingly unmanageable, the mental load contributed heightened stress (T20). Conversely, when logistical demands were manageable, perinatal women described feeling more in control and better able to cope with life’s stressors.

Planning for future events was a significant source of stress during both perinatal periods, particularly when there were social, environmental, or structural factors beyond their control that hindered their ability to manage responsibilities efficiently and effectively (T18). When available, social support helped offload logistical tasks, improving productivity (T21) and freeing up time for self-care (T22). Flexible scheduling enabled perinatal individuals to remain in the workforce longer and provided opportunities for women to invest in their health and responsibilities other than caregiving and domestic labor (T23). However, limited access to reliable childcare negatively impacted their physical and mental well-being, as they often had to sacrifice their own needs to meet competing demands associated with caregiving and work-related responsibilities (T24, T25).

Prioritizing others’ needs over their own was viewed as an inherent duty of motherhood, which began in pregnancy and intensified postpartum (T26). Prevailing social norms (e.g., traditional family structures, gender roles) and limited structural support, which shaped their perinatal experiences, reinforced this belief and were exacerbated by the pandemic (T27). In light of this belief, logistical demands commonly experienced during the perinatal periods were viewed to be a burden or an inconvenience to others, which left mothers feeling as if they needed to manage these demands alone. Ongoing attempts to manage these demands alone depleted physiological, psychological, and financial resources, which became increasingly difficult to sustain after childbirth (T28). With adequate support at the individual, organizational, and structural level, perinatal women reported they would experience relief from the mental load and stress associated with perinatal demands (T28).

#### Theme 3: Physical health

Physical health was the second leading barrier to mental well-being during pregnancy and third postpartum. Alternatively, it was the top facilitator to mental well-being during pregnancy and fourth postpartum. Their physical health concerns often centered on the health of others, likely in part as a result of the pandemic (T29). When they experienced physical limitations or medical complications during pregnancy or postpartum, it contributed to increases in stress and worry (T30). Conversely, when their physical health remained stable, they felt physically, mentally, and emotionally equipped to not only meet the demands of the perinatal periods but also engage in self-care to cope with the life’s stressors (T31).

The main physical health concerns were related to the pandemic, physical and psychological symptoms associated with pregnancy and postpartum, and the health and well-being of their children, support systems, and self. A major concern was the risk of contracting the COVID-19 virus and potential implications for their child, partner, broader social network, and self (T32). Pregnant individuals planning hospital births expressed significant stress and fear regarding the safety of the mother-child dyad and health of key support figures, with these feelings intensifying as their due date approached. (T32). Postpartum, when their physical health was prioritized and their concerns listened to rather than dismissed as “normal,” they no longer felt as if the physical challenges they were experiencing was something they “had to live with” (T33). However, for those reporting mental health challenges, their experiences were often perceived to benormalized and left unaddressed (T34).

Poor sleep quality and limited access to self-care were discussed in both perinatal periods but contributing factors differed between pregnancy and postpartum. During pregnancy, physical discomfort from pregnancy-related physiological changes made it difficult to stay asleep, which disrupted their ability to maintain pre-pregnancy routines and self-care routines (T35). Postpartum, they often described being in a persistent state of alertness (T36), hindering their ability to fall and stay asleep and reducing the energy needed for daily tasks and self-care. The child’s temperament and developmental stage also influenced sleep quality, either positively or negatively (T37). While maintaining pre-pregnancy routines and self-care during pregnancy was physically challenging, it became even more difficult following childbirth due to time constraints and the competing demands of newborn care, physical recovery from childbirth, and balancing work and domestic responsibilities. Many postpartum individuals expressed that there was barely enough time in the day to meet their basic needs (T38), leaving little to no time for engaging in self-care or health promotion activities (e.g., physical activity) (T39).

## Discussion

This study identified social support, logistical demands, and physical health as core determinants of stress during pregnancy and postpartum in a non-clinical, low-risk group of perinatal women. Importantly, these factors were perceived to be shaped by broader social and structural conditions, influencing perceptions of stress and coping. While these findings align with existing evidence [5, 13, 27, 28], much of the literature has examined these domains independently, and the strength and depth of evidence varies by domain. In contrast, this study offers an integrated perspective, highlighting dynamic interactions and period-specific similarities and differences among diverse determinants of maternal health and well-being that collectively shape maternal perceptions of stress and coping.

The postpartum period is increasingly referred to as the “fourth trimester,” reflecting growing recognition that physical and psychosocial demands extend beyond pregnancy and childbirth [5, 29]. Perinatal women in this study reported experiences consistent with the broader literature indicating the postpartum period is a particularly stressful period [30] but emphasized that stress experienced postpartum is interconnected with prenatal experiences, suggesting that perceptions of stress and coping evolve cumulatively across the perinatal continuum rather than independently within each perinatal period. The gradual accumulation of perinatal demands contributed to a steady rise in mental load, which were disruptive to their daily lives in ways reported outside of the context of a global pandemic. Resulting disruptions were initially subtle and manageable but intensified over time, becoming increasingly difficult to manage, especially as mother’s transitioned from pregnancy to postpartum, which brought about newly emergent and evolving demands. While these demands increased experiences of stress and the “mental load” [31], it was often not the presence of the demands that contributed to experiences of stress but the absence of reliable instrumental support at individual, social, and structural levels, particularly postpartum. Findings suggest that even among perinatal women presumed to have sufficient resources, they frequently perceived the resources available to them to be inadequate for meeting the cumulative demands of pregnancy and postpartum. This aligns with some qualitative findings in clinical populations [30], even in countries with more socially protective policies (e.g., paid parental leave, universal health insurance) [6] than the U.S., the country in which this study was conducted. Collectively, findings support multilevel interventions are needed, as improving the conditions in which routine perinatal demands are experienced may reduce the cumulative burden of stress across the perinatal continuum. Specifically, policies that strengthen social and structural support (e.g., affordable childcare, paid maternity leave, workplace accommodations, home-visiting programs) [32], alongside individual-level interventions (e.g., mindfulness-based interventions, mother-led support groups) [33], may better support women in navigating cumulative, competing demands across the perinatal continuum, thereby reducing stress and improving maternal health and well-being.

The steady accumulation of demands throughout pregnancy contributed to what perinatal women in this study referred to as the mental load, leading many pregnant participants to anticipate receiving increased support postpartum. Unmet expectations of increased support postpartum often resulted in heightened feelings of loneliness, which is consistent with some previously reported findings [34]. Notably, nearly all participants in the present study were married or at least lived with their partner yet still reported feeling lonely. This suggests that cohabitation or marital status does not necessarily equate to meaningful emotional or practical support, and for some, can even be an additional source of stress. Such findings highlight partner presence is likely an insufficient indicator of social support [35], underscoring the need for more nuanced assessments of support that consider quality, perceived adequacy, and types of support desired versus what is received. Similar to patterns reported for logistical demands, physical demands, particularly sleep, were described to have a comparable trajectory. Participants described sleep disruptions that began during pregnancy and progressively worsened as they approached their due date. For most, these disruptions intensified postpartum due to infant care needs, feeding schedules, and experiences of hypervigilance. The cumulative burden of these sleep disturbances directly contributed to stress, amplified the mental load, and reduced participant’s perceived capacity to cope with the persistent demands of the perinatal period.

Overall, findings illustrate how routine perinatal demands are not experienced as inherently stressful but are perceived to accumulate and become more or less manageable depending on the social and structural conditions in which they are experienced. Such findings may help explain why 3 out of 10 pregnant women and 4 out of 15 postpartum women screened positive for probable clinical depression, despite participants exhibiting clinical and sociodemographic profiles that would typically be categorized as low risk for complications. By exploring how contextual factors shaped maternal experiences of stress and coping, these findings provide insight into areas in which public health strategies and healthcare systems could make meaningful improvements to increase maternal support. Given chronic stress is a well-established risk factor for a range of maternal health complications [36, 37], prioritizing development and implementation of multilevel interventions aimed at reducing maternal stress may offer a high-value investment for maternal health promotion and prevention, aligning with global efforts to improve health outcomes among two of the world’s most vulnerable populations (i.e., perinatal women, children) [38]. While some stressors, such as sleep disturbances during pregnancy, might be difficult to address, others, like postpartum sleep disruptions due to newborn care, reflect responsibilities that could be redistributed or supported through formal support services. Similarly, work-place accommodations during pregnancy, such as schedule flexibility may reduce the burden of balancing occupational stressors with evolving physical and healthcare demands. Participants emphasized how physically demanding and psychosocially disruptive chronic sleep disturbances were, suggesting the potential value of structural level interventions to improve access to overnight postpartum care (e.g., nurse, midwife) [39]. Although implementation barriers remain, particularly in countries like the U.S., several nations (e.g., Sweden, Canada, Norway) have introduced policies and public health strategies aimed at promoting maternal recovery and reducing caregiver burden [40]. While further research is needed, findings suggest these models offer valuable examples of how structural supports can help reduce perinatal stress and promote maternal well-being during this vulnerable life-stage.

### Limitations

As a secondary analysis, data were collected to address the aims of the parent study, potentially limiting the depth of exploration in the present analysis. Data collection for the parent prospective cohort study occurred between 2019 and 2026; however, the qualitative interviews were conducted between 2020 and 2021 in response to research operations being interrupted by the COVID-19 pandemic, a globally shared event that rapidly reshaped the social and structural conditions that influence health and well-being. Given interviews were conducted over roughly 10 calendar months, they span multiple stages of the pandemic, influencing participant’s experiences and perceptions in ways that may not be reflective of experiences under non-pandemic conditions. While the pandemic may have exposed this low-risk cohort to conditions and challenges they may not have otherwise encountered to the degrees reported during the pandemic, a majority of the conditions and experiences described are not unique to the pandemic (e.g., pregnancy and childbirth related anxiety, competing logistical demands, occupational stressors), particularly for mothers belonging to marginalized groups (e.g., food access, healthcare access). Therefore, future research is needed to better understand how pregnancy and postpartum experiences are differentially shaped within and between diverse populations and settings.

## Conclusion

Findings from this study highlight that even among low-risk individuals, cumulative perinatal demands, particularly when compounded by insufficient support, can result in persistent stress exposures with potential implications for mental well-being. Social support, logistics, and physical health emerged as key factors contributing to or buffering against maternal stress, with access to these resources largely determined by social and structural factors beyond the individual’s immediate control. These findings underscore the need to account for broader social and structural support in perinatal health research and care. Future research and system-level changes are needed to address systemic inequities in perinatal care and promote the health of women, children, families, and the broader population.

## Data Availability

Unless otherwise determined by the PI of the parent study (EMW), de-identified data will only be shared on an as needed basis with collaborators directly involved in the study.

## Abbreviations

BDI: Beck Depression Inventory
EPDS: Edinburgh Postnatal Depression Scale
